# Comparisons of the Health Benefits of Strength Training, Aqua-Fitness, and Aerobic Exercise for the Elderly

**DOI:** 10.1155/2018/5230971

**Published:** 2018-06-19

**Authors:** Raquel Leirós-Rodríguez, Anxela Soto-Rodríguez, Ignacio Pérez-Ribao, José L. García-Soidán

**Affiliations:** ^1^Faculty of Physical Therapy, University of Vigo, Campus a Xunqueira, s/n, 36005 Pontevedra, Spain; ^2^Faculty of Nursing, University of Saint George, Campus Universitario Villanueva de Gállego, s/n, 50830 Zaragoza, Spain; ^3^Faculty of Education and Sport Sciences, University of Vigo, Campus a Xunqueira, s/n, 36005 Pontevedra, Spain

## Abstract

**Objective:**

To compare strength training, aqua-fitness, and aerobic exercise programs to discern the differences in the benefits achieved by each of the activities in older people.

**Design:**

Double-blind randomized trial.

**Setting:**

Controlled clinical environment.

**Participants:**

108 people: 54 female paired with a male of the same age (average age of 65.5 ± 5.6 years).

**Interventions:**

Three exercise programs (aqua-fitness, aerobic exercise, and strength training) for six months.

**Main Outcome Measures:**

Body Mass Index, Senior Fitness Test (which evaluated functional fitness), and the SF-12 Health Survey.

**Results:**

Men showed greater positive changes in the aerobic exercise group for general self-perceived mental health, leg strength, and flexibility of legs and arms. The largest improvements in overall self-perceived physical health and upper limb strength were in the men of the strength training group. The women participants in the strength training group obtained greater benefits, especially in self-perceived mental and physical health and in the strength of the four limbs.

**Conclusions:**

To maximise benefits, older people, in general, may want to consider participating in aerobic activity. Furthermore, older women would benefit greatly, both emotionally and physically, from exercise that includes strength training.

## 1. Introduction

The population is ageing, and more consideration needs to be given to the specific needs and problems of older people. The prevalence of overweight over 65 years old increases the risk of developing cardiovascular, metabolic, respiratory, and osteoarticular diseases [1, 2]. In addition, the ageing process modifies body composition (due to the loss of muscle mass and its progressive replacement by fat), which results in an increase in body mass index (BMI) (an indicator of morbidity, dependence, and mortality) in adults and elderly people [3]. Consequently, functional decline increases sanitary and assistance economic expenditure [4–7]. A sedentary lifestyle exacerbates these symptoms and the progressive loss of autonomy and quality of life of the elderly [8, 9].

From the age of 65, about 40% of people will suffer from a loss of some type of dependence, particularly in terms of physical dependence [10, 11]. There is an increasing burden of musculoskeletal disorders among sedentary and ageing populations. Musculoskeletal conditions can impair a person's ability to undertake physical activity. The key step in the development of effective intervention strategies to assist inactive patients with musculoskeletal disorders to become more physically active is to understand their perceived barriers to undertaking physical activity in their daily lives. Respecting their preferences for types of exercise while adapting to the schedule of individual exercise sessions is key to ensuring their adherence to exercise programs [12].

Naci and Ioannidis [13] highlighted the therapeutic value of physical activity, which reduces mortality for many common health conditions at rates that are similar to those of pharmaceutical treatments. In addition, physical activity is essential for healthy ageing. It has been shown, when in the form of structured exercise, to have a positive effect on gait performance, balance control, muscular strength, cardiovascular fitness, and quality of life.

In light of the benefits of physical activity, many physical-activity programs are encouraged by institutions and public health professionals [5, 14, 15]. However, there is heterogeneity in the different physical-activity programs that are offered to the elderly. Although guidelines for the performance of physical activity by elderly people have previously been described [16, 17], they are general and they have not been described in terms of the specific needs of the individual.

For the purposes of this paper, we focused on three activities that are associated with beneficial effects for the physical and psychosocial well-being of the elderly. First, strength training programs promote strength, prevent osteoporosis, and osteopenia, facilitate the achievement of activities of daily living (ADL), improve posture, and slow the weakening of muscle fibres that occur as a result of ageing [18, 19]. Second, aqua-fitness activities have been shown to reduce feelings of fatigue (especially during the performance of ADL), decrease fat percentage, improve coordination, and balance and facilitate socialisation [20–22]. Third, aerobic exercise classes have an ability to improve cardiovascular status, limb strength, limb and trunk joint range, coordination, and balance [20, 23–25].

On the basis of the benefits described above, this study compared strength training, aqua-fitness, and aerobic exercise to discern the differences in the benefits achieved by each of the activities in older people.

## 2. Material and Methods

### 2.1. Sample

**Figure 1:**
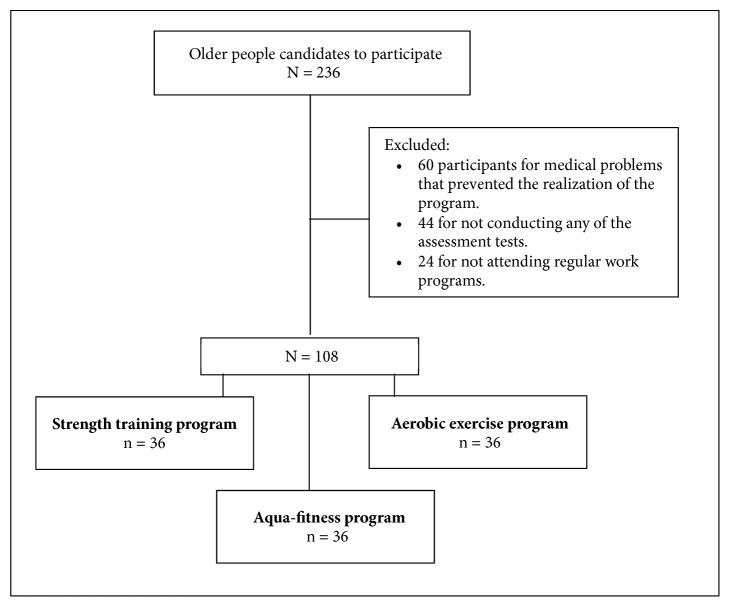
CONSORT flow diagram.

The sample consisted of 108 people (54 female participants paired with a male of the same age) between 50 and 79 years old (average age of 65.5 ± 5.6 years) (Figure 1). The participants were divided into three activity groups, as shown in Table 1. Each participant was in a physical activity and health promotion program that was created for the purposes of this research. Participation was voluntary throughout the course of the study, and the choice of different activity groups was randomized.

All participants gave informed consent, in accordance with the Helsinki Declaration (rev. 2008). This research received ethical approval from the Commission of Ethics of the Faculty of Sciences of Education and Sport of the University of Vigo (Spain) (code: 3-0504-16).

### 2.2. Procedure

Evaluations were performed using the Rikli and Jones (1999) Senior Fitness Test (SFT), which evaluated functional fitness, and the SF-12 Health Survey that measured quality of life in relation to health. During the investigation, three data-collection points were set.

(i) Initial test: prior to the start of the program. A doctor checked the health status of the participants and their possible limitations or contraindications to the performance of any of the program's activities. Then, anthropometric measures of height, weight, and BMI were taken. Finally, the SFT and the SF-12 questionnaire were applied to the participants.

(ii) Intermediate test: at 12 weeks after onset. The same measurements were taken as at the beginning, except for the medical interview.

(iii) Final test: at 24 weeks. It is identical to the intermediate test.

All the evaluations were carried out by monitors and graduates in physical therapy, previously instructed and trained for these procedures. All participants were randomized into three groups. The different programmes were given by monitors and graduated in physical therapy and lasted six months.

### 2.3. Activity Groups

#### 2.3.1. Strength Training Program

Participants in the strength training group had a mean age of 66.1 ± 4.3 years. The strength programme progressed according to the tests performed by the participants. During the first six weeks, the sessions consisted of an initial warm-up time of ten minutes, performing general mobility exercises and stretching. This was followed by directed bodybuilding activity of 30-40 minutes, which was progressively taught, assimilating the functioning of each muscle machine. A circuit of eight exercises that worked the large muscle groups of the upper and lower extremities was performed comprising two sets of twelve repetitions with loads of 50-60% of the maximum resistance (MR). The ten final minutes were devoted to stretching of the muscle groups worked during the session. From the seventh week, loads increased, and MR tests were performed to find out the individual work percentages. The work programme was modified in the seventh week, to ten repetitions at 60% MR; then the repetitions were increased to twenty at weeks seven and eight. Finally, during the last four weeks, participants completed circuits of three series between 60 % and 80% of MR, with ten repetitions per set and two-minute breaks. This training session was equivalent to 2 METS.

#### 2.3.2. Aqua-Fitness Program

Participants in the aqua-fitness group had a mean age of 66.1 ± 4.9 years. They were divided into classes of ten to twelve people. The pool had a depth of 1.40 metres in the central area and 1.75 metres at the end. The classes lasted 55 minutes. The timing of this activity comprised an initial period of two weeks of low intensity; this period served to evaluate the average level of each group. They then went on to an improvement stage, where the repetitions and then the intensity were progressively increased between weeks three to twelve. The basic structure of the sessions consisted of five initial minutes of joint mobility and warm-up, aerobic work (25 minutes), strength-resistance work (ten minutes) during which the body regions targeted varied according to the purpose of the session (chest, shoulder and dorsal region, arm and forearm region, lower limbs and muscles of the abdominal region), games (ten minutes), and stretches (five minutes). This training session was equivalent to 3 METS.

#### 2.3.3. Aerobic Exercise Program

Participants in the aerobic exercise group had a mean age of 64.3 ± 7.1 years who were divided into groups of 15, to be able to perform the sessions. These lasted for 55 minutes, with a minimum warm-up time of ten minutes and five minutes of stretches at the end. All sessions also included a central component of 40 minutes which mainly comprised choreographed aerobic exercises. Eventually, some strengthening exercises with autoloads for the large muscle groups were performed. This training session was equivalent to 3 METS.

### 2.4. Data Analysis

The SPSS statistical package (version 22) was used to analyse the data. The variables showed a normal distribution according to the Kolmogorov-Smirnov test (p > 0.05), and the variances were homogenous, as determined by the application of the Levene test. The t-test for related samples compared the evolution of each activity group throughout the program. To compare the effect of the different activity groups, an analysis of variance was used with a Bonferroni correction. The level of significance was set at p < 0.05.

## 3. Results

### 3.1. Results by Activity Group

The complete results are shown in Table 1.

The participants in the strength training group showed statistically significant differences in the SFT. Progressive improvements were identified from the beginning of the program for leg strength (p = 0.02), arm strength (p = 0.04), and flexibility, especially arms (p = 0.03). In addition, significant improvements in weight (with an average reduction of 2 kg per person) and BMI (p < 0.001) were observed. In addition, results from the SF12 questionnaire showed significant changes for pain and general health (p = 0.02 for both).

The participants in the aqua-fitness group demonstrated improvements in all skills measured by SFT (p values between 0.03 and 0.01 in all four variables) and, to a lesser extent, in weight (p = 0.03) and BMI (p = 0.04). With regard to the SF12 questionnaire, this group showed improvements in pain (p = .004), physical function (p = 0.03), emotional limitations (p < 0.001), and general and mental health (p = .002 for both).

In the aerobic exercise group, significant improvements were observed for the skills measured by SFT, including the strength and flexibility of all four limbs, particularly arm strength (p < 0.001). Weight and BMI were also significantly reduced (average loss of 4 kg per person; p < 0.001. Results from the SF12 questionnaire demonstrated significant positive results for emotional limitations and physical function (p < 0.001) and, to a lesser extent, for pain and general health (p = 0.02 and 0.01, respectively).

### 3.2. Results by Sex

**Figure 2:**
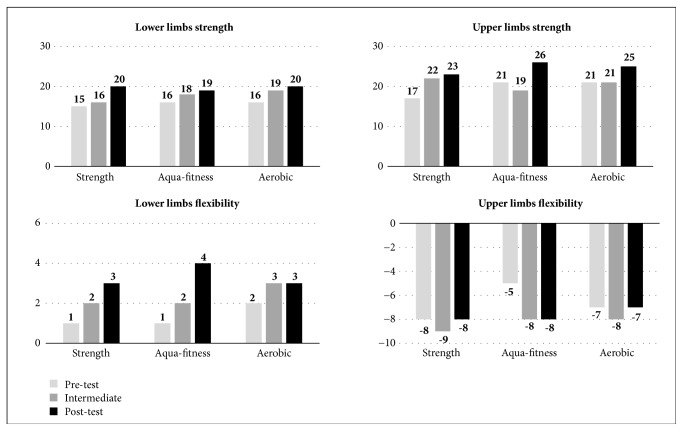
Senior Fitness Test women's results by activity program.

The men showed greater positive changes in the aerobic exercise group (Figure 2). For this activity, noticeable benefits were observed for general self-perceived mental health, leg strength, and flexibility of legs and arms (p values ranged between 0.001 and 0.02 in the four variables). For the aqua-fitness group, the smallest improvements were obtained in general mental health and strength of legs and arms (with changes 50% lower compared to the aerobic exercise group). However, the largest improvements in overall self-perceived physical health and upper limb strength were in the strength training group (p = 0.01 for both).

**Figure 3:**
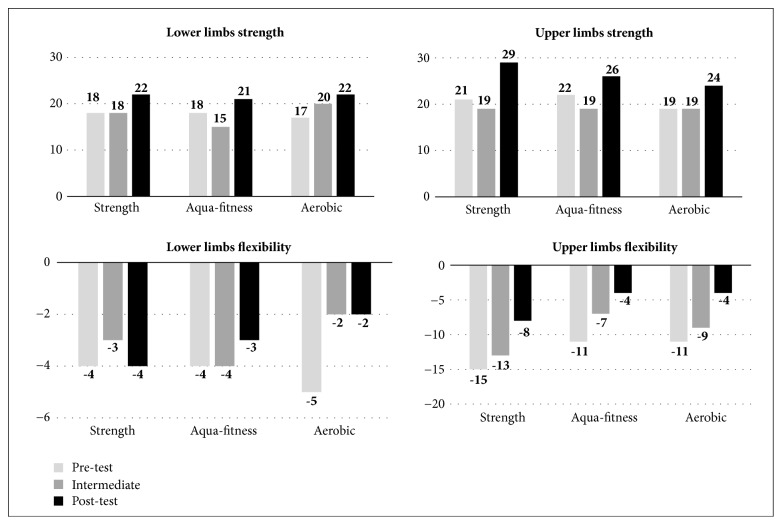
Senior Fitness Test men's results by activity program.

The women participating in the strength training and aerobic exercise benefited the least during the duration of this research study (Figure 3). The strength training and aerobic exercise programs produced the most improvements for the strength and flexibility of the four limbs and self-perceived mental health. In contrast, the participants in the strength training group obtained greater benefits, especially in self-perceived mental and physical health and in the strength of the four limbs (p values between 0.02 and 0.001 for the four variables).

## 4. Discussion

The objective of the study was to compare three physical activity programs (strength training, aqua-fitness, and aerobic exercise) in order to discern the differences between the benefits achieved by each for people aged 50 years and older.

Regarding the specific improvements as a result of each program, the aerobic exercise program was markedly beneficial for weight loss and reduced BMI, and these benefits were observed earlier compared to the aqua-fitness and aerobic exercise. In addition, the participants reported improvements in emotional limitations, physical function, pain, and general self-perceived health (variables estimated by the SF-12 questionnaire). All of these improvements are in line with recommendations to promote aerobic exercise training during ageing because it has already recognised benefits for functionality, emotional health, and blood glucose control [26, 27].

The aqua-fitness program produced less pronounced improvements in the functions evaluated by the SFT. For example, no significant changes were observed in the strength of lower limbs during or after the program. However, it was the activity that resulted in the most improvements in self-perceived mental health. It was matched with aerobic exercise in terms of emotional limitations, pain, and general health. These results agree with those obtained by Vécseyné-Kovách et al. [21], as well as in the small improvement in weight and BMI obtained by the participants in this activity.

The strength training program obtained inferior results for the SFT variables compared to the aerobic exercise program. These results contradict the previous study by Cadore et al. [18], in which he stated that strength training performed at a moderate intensity in older populations is the most effective way to improve neuromuscular and cardiorespiratory functions. In the present study, the strength training program was not as effective as the other programs studies.

In the gender-differentiated analysis, the results for the males are comparable to those of the sample as a whole—that is, large benefits were observed following the participation in aerobic exercise, and somewhat smaller benefits were observed following participation in strength training activities and the smallest benefits were observed following participant in aqua-fitness.

The analyses of the females changed this sequence such that strength training was the activity that showed the greatest improvement. This phenomenon may be due to the fact that, in men, the process of muscle mass loss is not as pronounced as in postmenopausal women, who are susceptible to hormonal changes that can produce sarcopenia [2], so the men did not benefit as much from the muscle empowerment work. These results concur with the recommendations of introducing muscular empowerment training in fragile elders in order to improve the general physical state of this population and to prevent disability [18]. This is especially important for the women for their longer life expectancy [11].

This study clearly highlights a need for the implementation of correctly designed interventions with the goals of increasing the participation of older adults, improving physical health, and preventing age-related impairment. Previous studies that have been focused on the relationship between regular physical activity and healthy ageing, its effects on physical performance, and cognitive and psychological function in older adults have been based on intervention programs [14, 17, 28]. However, this study is the first to compare three activities in both sexes over a long period (6 months). There are some limitations, including the lack of data about the changes in body composition that occur following participation in each of the programs and the monitoring of the programs over an extended period of time and that we must take into account for future research.

The study participants had a high degree of adherence to the exercise programs. This may have been the result of the professionalism of the graduates and monitors and the adequate design of the training sessions.

## 5. Conclusions

All participants showed improvements in physical and psychological variables during and after the three exercise programs. We conclude that physical activity programs that are specifically aimed at older people should take into account the initial physical condition of the participants. To maximise benefits, older people, in general, may want to consider participating in aerobic activity (such as aerobic exercise). Furthermore, older women would benefit greatly, both emotionally and physically, from exercise that includes strength training.

## Figures and Tables

**Table 1:** Distribution and results of the participants according to the different activities.

	**ALL** (n = 108)		**STRENGTH PROGRAM** (n = 36)		**AQUA-FITNESS** **PROGRAM **(n = 36)		**AEROBIC EXERCISE PROGRAM **(n = 36)	
	**Pre-test**	**Post-test**	**Pre-test**	**Post-test**	**Pre-test**	**Post-test**	**Pre-test**	**Post-test**
**Weight**	65.8 ± 8.6	63 ± 6.1*∗∗*	64.3 ± 8.4	62.2 ± 4*∗∗*	68.8 ± 3.9	67.6 ± 6.1*∗*	65.6 ± 11.2	61.8 ± 8.3*∗∗∗*
**Body Mass Index**	28.4 ± 4.1	26.6 ± 4*∗∗*	27.6 ± 4.1	25.9 ± 3.3*∗∗∗*	30.1 ± 3.1	29 ± 4.6*∗*	28.2 ± 4.7	26.1 ± 4.3*∗∗∗*

**Rikli & Jones (Senior Fitness Test):**								

**Strength Lower Limbs**	17.2 ± 5	20.6 ± 4.7*∗∗∗*	16.7 ± 3.6	19.8 ± 4.7*∗*	19.5 ± 5.9	21.3 ± 4.7	15.9 ± 5.1	20.8 ± 4.9*∗∗*
**Strength Upper Limbs**	20.9 ± 5.7	25.9 ± 5.7*∗∗∗*	21.1 ± 6.2	26.9 ± 6.3*∗*	21.8 ± 5.2	27.1 ± 5.5*∗∗*	20.1 ± 5.7	23.9 ± 4.7*∗∗∗*
**Flexibility Lower Limbs**	-1 ± 8.2	-0.9 ± 9*∗*	-1.5 ± 8.6	-1.3 ± 8.9*∗*	0.1 ± 7	0.9 ± 9.1*∗∗*	-1.5 ± 9	-0.5 ± 9.3*∗*
**Flexibility Upper Limbs**	-9.2 ± 10.9	-7.4 ± 10.4*∗∗∗*	-11 ± 10.8	-8.1 ± 11.9*∗∗*	-6.5 ± 11.3	-5.1 ± 8.9*∗∗*	-9.4 ± 10.8	-7 ± 10.3*∗*

**Short Form Health Survey (SF12):**								

**Physical function**	51.9 ± 7.7	54.5 ± 5.7*∗∗∗*	53.5 ± 5.8	54.7 ± 5.3	51 ± 8.2	53.3 ± 7.3*∗*	51.9 ± 8.6	54.6 ± 5.9*∗∗∗*
**Physical limitations**	39 ± 1.5	39.1 ± 1.7	39.1 ± 1.2	38.7 ± 1.7	39 ± 1	39.4 ± 1.6	38.9 ± 2	38.2 ± 1.9
**Pain**	33.4 ± 14.1	26.5 ± 11.5*∗∗∗*	29.3 ± 12.4	26.2 ± 11.5*∗∗*	35.7 ± 15.2	29.6 ± 13*∗∗*	34.9 ± 15	25 ± 10.4*∗∗*
**General health**	41.1 ± 9.7	47.1 ± 9*∗∗*	43.2 ± 9	47 ± 9.4*∗∗*	38.4 ± 11.2	46 ± 9.2*∗∗*	39.3 ± 8.7	47.3 ± 8.8*∗∗*
**Vitality**	40.7 ± 11.7	44.7 ± 9.1*∗*	38.7 ± 9.1	38.2 ± 7.9	37.2 ± 12.2	37.9 ± 10.6	45.6 ± 12.4	45.7 ± 9.1
**Emotional limitations**	54.3 ± 7.6	53.8 ± 4.5*∗∗∗*	56.6 ± 0	56.6 ± 0	52.9 ± 9.6	50.1 ± 2.2*∗∗∗*	53.7 ± 8.6	50.8 ± 7.8*∗∗∗*
**Social functions**	20.7 ± 3.8	21.4 ± 3.2	21 ± 3.6	21.4 ± 3.1	20.8 ± 3.6	20 ± 2.8	21.5 ± 2.7	21.9 ± 3
**Mental health**	49.4 ± 8.3	49.3 ± 7	49 ± 9.4	49.1 ± 3.9	50.4 ± 9.1	52 ± 6.7*∗∗*	49.7 ± 6.1	49.3 ± 7.9
**General physical health**	44.9 ± 4.6	45.2 ± 4.7*∗*	44.8 ± 4.7	45.5 ± 4.5	44.2 ± 4.3	45.8 ± 5.8	44.8 ± 4.2	44.9 ± 4.8
**General mental health**	38.6 ± 5.2	38.4 ± 4.6	38.7 ± 5.8	38.2 ± 4.1	38 ± 5	38.4 ± 4.3	40 ± 5	40.2 ± 5.7

*∗*p < 0.05; *∗∗*p < 0.01; and *∗∗∗*p < 0.001.

## Data Availability

The data used to support the findings of this study are available from the corresponding author upon request.
